# Color patterns as a model for connecting developmental mechanisms to phenotypic evolution

**DOI:** 10.1038/s41467-026-75406-w

**Published:** 2026-09-17

**Authors:** Hannah I. Weller, Claudius F. Kratochwil, Merlijn Staps

**Affiliations:** 1 Helsinki Institute of Life Science (HiLIFE), University of Helsinki, Helsinki, Finland; 2 Faculty of Biological and Environmental Sciences, University of Helsinki, Helsinki, Finland; 3 Department of Ecology & Evolutionary Biology, Princeton University, Princeton, NJ USA; 4 Present Address: Department of Biology, Washington University in St. Louis, St. Louis, MO USA

**Keywords:** Evolutionary developmental biology, Biodiversity, Pattern formation

## Abstract

Vertebrate color patterns are experimentally accessible and ecologically relevant traits, making them excellent models for studying how diversity arises during development and evolution. Advances in developmental biology, pattern quantification, and broad phylogenetic sampling now offer an opportunity to investigate how pattern development and evolution influence each other. To facilitate this crosstalk, we propose a conceptual framework connecting pattern development with its diversification through two key developmental principles: self-organization and instruction. This integrative framework advances our understanding of color pattern evo-devo and provides a roadmap for connecting development with diversification across traits and taxa.

## Abstract (web summary)

Color patterns are not only visually striking but also a compelling model trait for bridging evolution and development. This perspective outlines how mathematical modeling and state-of-the-art phenotyping, interpreted through developmental mechanisms, can guide hypothesis testing in evo-devo research.

## Introduction

### The development and evolution of color patterns

A major goal of evolutionary developmental biology is to understand how evolutionary and developmental processes interact to generate biodiversity^1–5^. This includes how developmental mechanisms evolve to generate new forms in response to selection, but also how development facilitates or constrains diversification by influencing what forms can be generated (or are more likely to be generated). Investigating the interplay between evolution and development requires study systems where we can access both the taxonomic breadth of phenotypic diversity and the mechanistic depth of its molecular-developmental underpinnings.

**Fig. 1:**
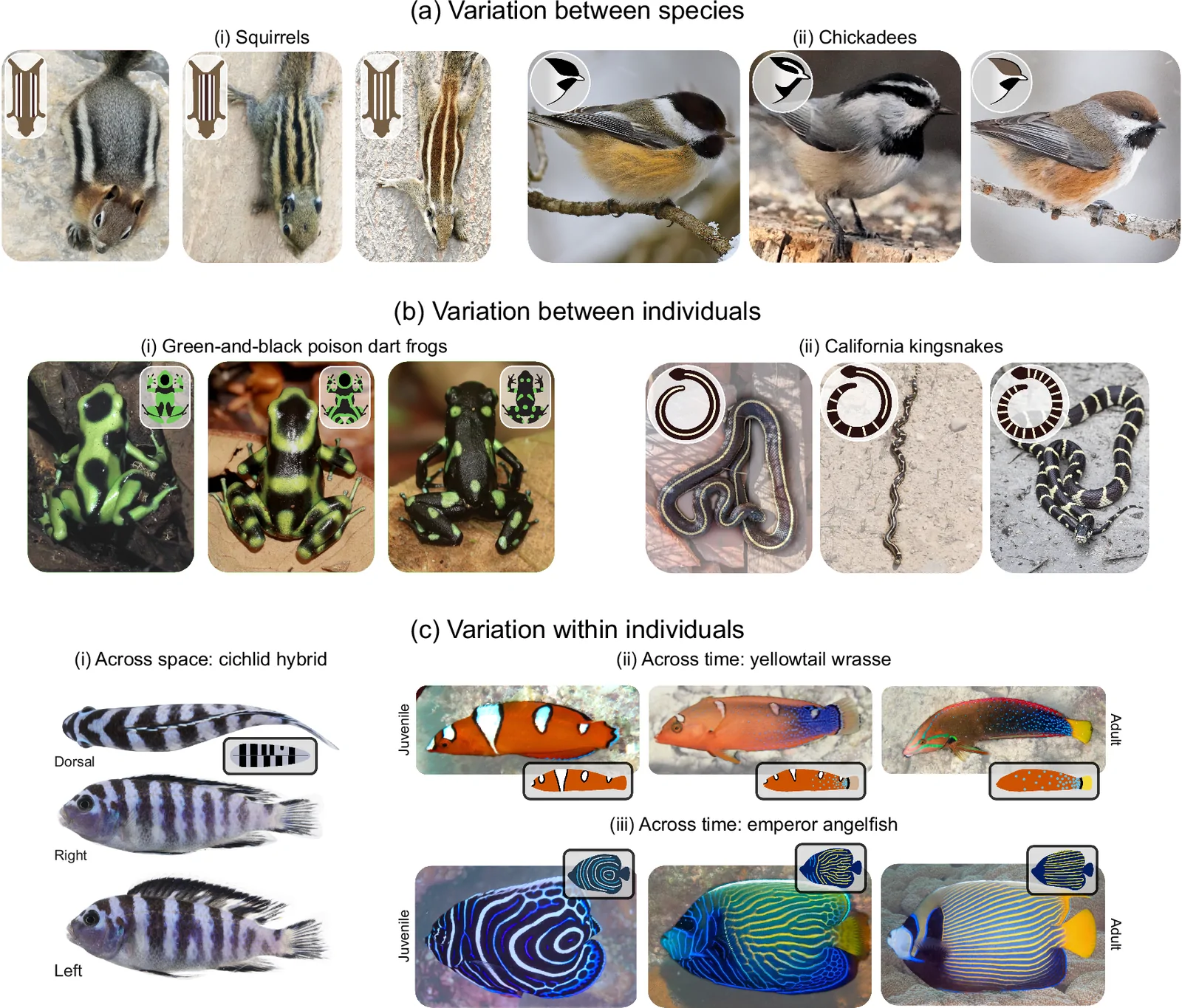
Examples of vertebrate pattern diversity across biological scales. **a** Pattern variation between species in a clade, such as squirrels (Sciuridae, i) or chickadees (*Poecile* spp., ii). Species shown: common golden-mantled ground squirrel *Callospermophilus lateralis* (photo credit T. Folsom, CC-0), Himalayan striped squirrel *Tamiops macclellandi* (S. Hambly), five-striped palm squirrel *Funambulus pennantii* (iNaturalist user saydelah, CC-BY), black-capped chickadee *P. atricapillus* (A. Konings), mountain chickadee *P. gambeli* (P. Hardy, CC-BY), chestnut-backed chickadee *P. rufescens* (G. Beaton). **b** Pattern variation between individuals of the same population, such as (i) the polymorphic green-and-black poison dart frog *Dendrobates auratus* (photo credits iNaturalist user geosesarma; P. Uetz; H. Raney, all CC-BY) and (ii) California kingsnake *Lampropeltis californiae* (V. Scheidt; B. Wright; iNaturalist user nmoorhatch, CC-BY). **c** Pattern variation within individuals, which can manifest (i) across space, such as left-right asymmetries in a hybrid cross of cichlid fish *Chindongo demasoni* × *Chindongo saulosi* (H.I. Weller) or across time, as in (ii) the shift from white bars to blue spots in the maturing yellowtail wrasse *Coris gaimard* (iNaturalist user portioid; R. Rittmaster; D. Loarie; all CC-BY) and (iii) the shift from concentric rings to stripes in the emperor angelfish *Pomacanthus imperator* (D. Delso, delso.photo, CC-BY-SA; S. & J. Johnson; R. Zerpe, CC-BY). Copyright licenses: https://creativecommons.org/licenses/by/4.0/, https://creativecommons.org/licenses/by-sa. Photos were cropped, and minor cosmetic adjustments (e.g., fading out the background) were made.

Vertebrate color patterns are emerging as just such a system. Many vertebrates exhibit spatial variation in pigmentation and/or structural colors across the integument (e.g., skin, fur, feathers, and scales) that can provide insights into tissue patterning on a developmental scale^6^. Color patterns also exhibit striking diversity in pattern organization across levels of biological organization^7–14^ (Fig. 1). Importantly, pattern diversity is accessible non-invasively and can be feasibly characterized at a broad scale, especially given recent progress in high-throughput phenotyping, computational image analysis, and digitization of museum collections^15–17^. On the other hand, the developmental mechanisms by which color patterns arise during development have long remained obscure, particularly outside of traditional model organisms. Recent advances in molecular and sequencing techniques have enabled researchers to investigate the molecular and genetic basis of color patterns in increasing depth^6^, and by now, the list of species in which developmental studies of pattern formation have been conducted spans major vertebrate groups, including squamates^18,19^, fishes^20–22^, birds^23–27^, and mammals^28–30^.

A central challenge in connecting pattern evolution and development is that development is typically studied at the molecular level in only a small number of model species, but making robust inferences about diversification requires research on whole clades. As a result, developmental and evolutionary studies have often proceeded independently, with developmental studies elucidating various mechanisms that govern the formation of specific “model” patterns (e.g., those of zebrafish^22^ and striped mice^29^), and evolutionary studies describing how patterns diversify across many species (e.g.,^31–36^). Recent years have seen exciting developments at the interface between pattern development and evolution: researchers have probed the mechanistic underpinnings of pattern variation in polymorphic species (e.g., Fig. 1b)^19,37–42^ and among closely related species^24,37–39^, and invoked developmental principles to explain patterns of diversification^43–45^. These works highlight the potential of using color patterns to bridge development and evolution and underline the need for overarching frameworks that provide a fundamental understanding of how changes in pattern development link to changes in pattern organization at evolutionary scales.

Here, we propose a general framework for connecting pattern development with pattern diversification that is designed to facilitate the integration of developmental and evolutionary studies of color patterns. We focus on two fundamental principles of pattern formation: (1) self-organization, where spatial organization emerges endogenously from local processes (e.g., cell–cell interactions), and (2) instruction, where spatial organization is imposed by exogenous cues. We first review how these two abstracted mechanisms, and the interactions between them, can broadly explain pattern development across different systems. We then explore their relative roles in pattern evolution and formulate general predictions for the developmental basis of pattern variation. Finally, we illustrate how this conceptual understanding yields concrete ways to integrate pattern development and evolution in practice, by generating developmental hypotheses from observed pattern variation and by testing how development biases pattern diversification. Altogether, we provide a roadmap for color pattern evo-devo that integrates developmental insights, surveys of cross-species pattern diversity, and mathematical models for pattern formation.

### Principles of pattern development

**Fig. 2:**
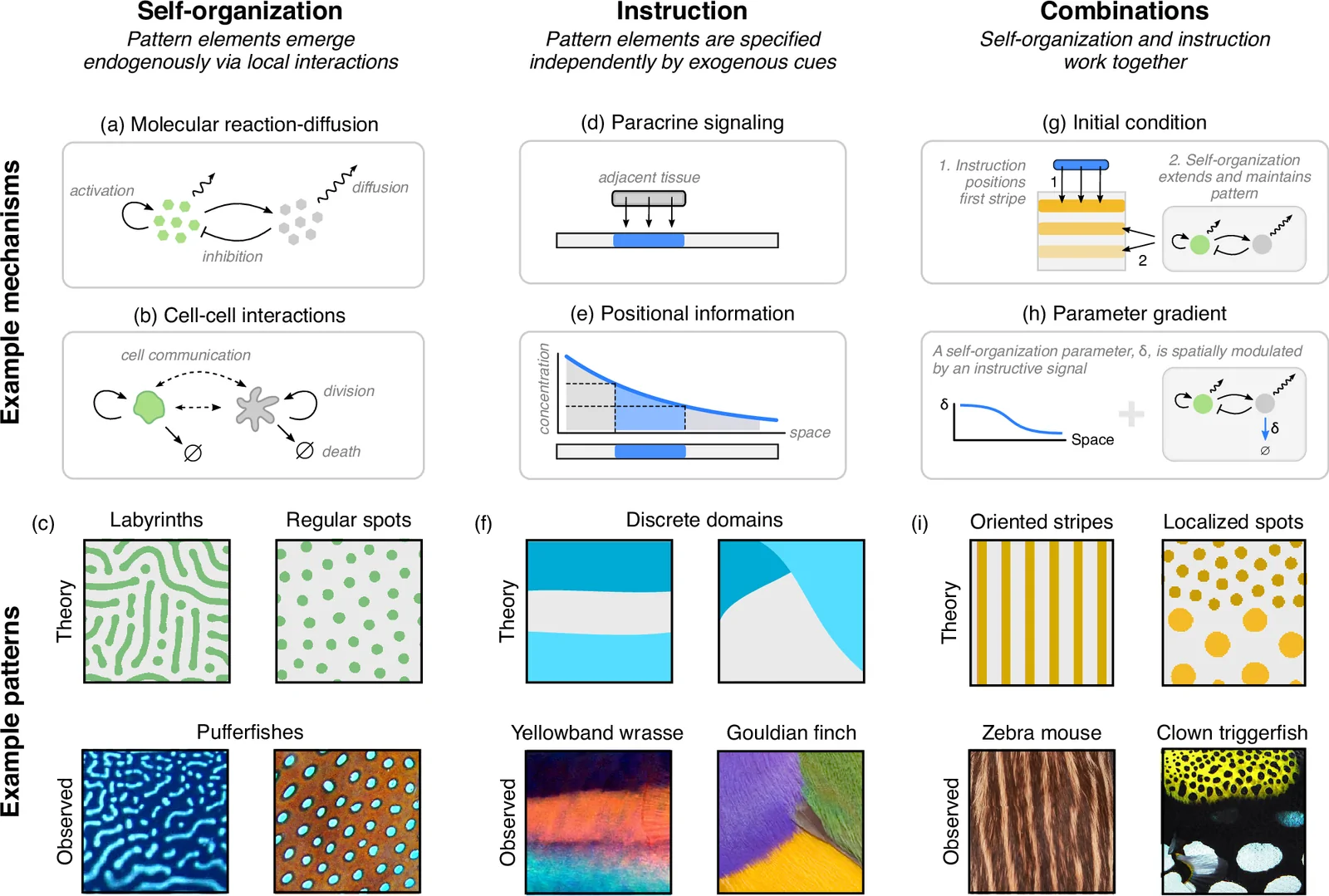
Self-organization and instruction in pattern development. Mechanisms for pattern self-organization include, for example, reaction-diffusion of an activator and inhibitor molecule (**a**) or long- and short-range interactions between different cell types (**b**). **c** Self-organization can produce labyrinthine or spot patterns, similar to those of the map pufferfish *Arothron mappa* (photo credit M. Baum, CC-BY-SA) and spotted sharpnose puffer *Canthigaster solandri* (N. Hobgood, CC-BY-SA). Instructive mechanisms include, for example, paracrine signaling (**d**), in which signals from adjacent tissue define pattern elements, or morphogen gradients that provide positional information (**e**). **f** Instruction produces patterns with discrete domains, as observed in yellowband wrasse *Cirrhilabrus luteovittatus* (S. & J. Johnson) or Gouldian finch *Chloebia gouldiae* (M. Pot, CC-BY-SA). **g** Combination example in which the first stripe of a pattern is defined by instruction and additional stripes self-organize. **h** Combination example in which an instructive signal spatially modulates one of the self-organization parameters (the death rate δ of the gray cell type). **i** Combinations of self-organization and instruction can, for example, produce oriented stripes, as in zebra mouse *Lemniscomys zebra* (M. Staps), or localized spots, as in clown triggerfish *Balistoides conspicillum* (H. Zell, CC-BY-SA). Copyright licenses: https://creativecommons.org/licenses/by-sa. Photos were cropped and rotated.

Pattern development can be described phenomenologically in terms of two general principles: self-organization and instruction (Fig. 2). Self-organization broadly describes the endogenous formation of patterns via local interactions. By contrast, we use the term instruction to refer to pattern formation mechanisms, including positional information, where pattern elements are specified by exogenous cues. Both principles were initially proposed by theoreticians, but their generality has since been confirmed by accumulating empirical and experimental evidence across different systems.

The idea of pattern self-organization was famously formalized in the 1950s by Alan Turing, who showed mathematically that the interactions between two diffusing molecules, an activator and an inhibitor, could give rise to emergent patterns^46^ (Fig. 2a). The key characteristic of the resulting “Turing patterns” is repeating pattern elements (e.g., spots, stripes, or labyrinthine bands), whose exact position is random, but whose relative spacing is highly consistent. Because these features are reminiscent of the color patterns of real organisms (e.g., the pufferfishes in Fig. 2c), Turing’s work inspired mathematical biologists to build numerous variants of his initial model to recapitulate the periodic color patterns of various animals, including zebras, big cats, and snakes^47,48^. While it might not necessarily be surprising that almost any biological pattern could, in principle, be reproduced by a sufficiently complex mathematical model, the striking feature of Turing’s model is that it recapitulates a broad diversity of naturally occurring color patterns from simple principles.

Theoreticians realized that self-organized pattern formation requires two key components: short-range activation, which establishes individual pattern elements, and long-range inhibition, which creates the gaps between neighboring pattern elements^49–51^. The coupling of short-range activation and long-range inhibition can arise at different levels of biological organization (e.g., molecular or cellular) and by different mechanisms (e.g., chemical or mechanical). For example, rather than arising from molecular interactions between an activator and an inhibitor, as in Turing’s original proposal, short-range activation and long-range inhibition can also emerge from contact-mediated communication between cell types across different distances^52^ (Fig. 2b) or from the bending and buckling of a growing tissue^53^.

Early evidence for self-organized color pattern formation came from the observation that the dynamic changes in pattern organization in the emperor angelfish *Pomachanthus imperator* (see Fig. 1c) are consistent with what would be expected from reaction-diffusion models^54^. Experimental evidence for pattern self-organization was subsequently obtained in zebrafish by ablating part of the pattern using lasers and then observing how the pattern dynamically reorganized in the ablated region, maintaining the spacing but not the orientation of the original pattern^55^. Pattern formation in zebrafish involves three types of pigment cells: melanophores, xanthophores, and iridophores. Extensive experimental work has elucidated the cellular interactions between these cell types and shown that they are required for successful pattern formation^22,38,55–59^. Conversely, these experiments have been complemented by mathematical models showing that the cellular interactions are, at least in theory, sufficient to explain pattern formation^52,56,60,61^, even if these models do not capture the full complexity and redundancy of the actual biology. While fish provide arguably the best-studied example of self-organized pattern formation, self-organization has also been implied in the formation of periodic feather patterns in birds^25,26,62^.

In instruction, pattern elements are specified by an exogenous cue rather than emerging from local interactions. Possible cues include prepatterns, anatomical structures serving as scaffold or guidance, paracrine signals (Fig. 2d), or molecular gradients (Fig. 2e). For example, the idea that molecular gradients can provide a developing tissue with positional information was proposed by Lewis Wolpert^63–65^. He illustrated this idea with the toy example of a “French flag” pattern, which would form if cells in a developing tissue would adopt red, white, and blue identities according to their position along a pre-established concentration gradient of a molecule Wolpert referred to as a morphogen^63^. Unlike in self-organization, instructive mechanisms define pattern elements independently; moreover, the location of these pattern elements can be highly consistent because it is under direct control of the instructive cue rather than emerging endogenously. This makes instruction an attractive candidate to explain the formation of patterns consisting of discrete domains with boundaries in consistent locations (Fig. 2f).

There is substantial empirical evidence for the role of instructive cues in vertebrate pattern formation. For example, the paracrine factor Agouti controls countershading in both mammals^28^ and fish^66^, by inhibiting melanization ventrally. In juvenile galliform birds, the formation of stripes depends on cues from the somitic mesoderm^24^. And in the pearl danio fish, a gradient of the morphogen BMP provides positional information in the fin, controlling a shift from red to yellow pigment cells along the proximal-to-distal axis^67^. Thus, there is extensive experimental evidence for the generality of instruction and self-organization across different vertebrate systems, even if the underlying molecular mechanisms vary.

While self-organization and instruction were initially viewed as competing alternatives, they often work in concert. Specifically, instructive cues can influence the subsequent self-organization of a pattern, for example, by providing an initial template that provides a starting point for self-organization to elaborate on (Fig. 2g), or, alternatively, by spatially modulating the local interactions underlying self-organization (Fig. 2h). Combining self-organization with instruction expands the space of possibilities to include various complex, yet robust patterns^68,69^. For example, while self-organization alone may lead to intricate labyrinthine patterns with random orientation (Fig. 2c), the incorporation of an instructive signal can be sufficient to orient the pattern and generate stable stripe patterns with a consistent orientation, as in rodents (Fig. 2i)^45,70^ and zebrafish^22,61,71^. Similarly, instruction can restrict self-organized patterns to particular regions of the skin by defining the domains in which self-organization can act. This mechanism can be invoked to explain abrupt changes between different types of periodic patterns, such as from small, black spots on a yellow background to big, white spots on a dark background in clown triggerfish (Fig. 2i).

### Linking pattern development and pattern variation in theory

**Fig. 3:**
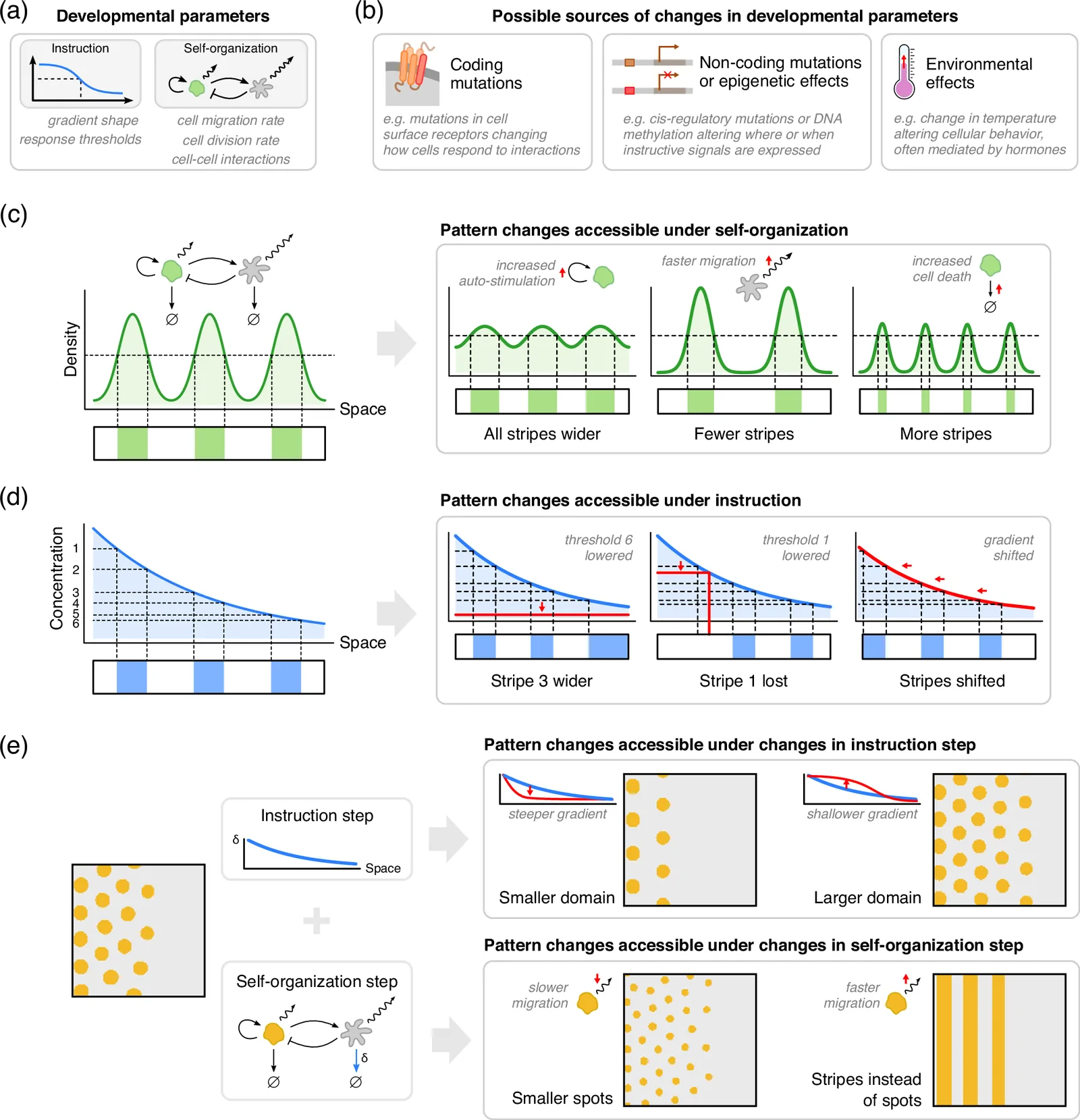
Changes in pattern development and their consequences for pattern organization. **a** Example developmental parameters influencing pattern organization for instruction (left) and self-organization (right). **b** Changes in developmental parameters depend on both genetic and environmental factors. **c** A one-dimensional pattern of three equidistant domains can be generated by self-organization (here shown as the interactions between two migrating cell types). In this case, possible pattern variants include global changes in domain width and number. **d** The same pattern can also be generated by instruction (here shown as interpretation of a morphogen gradient). Possible variants now include changes to individual domains and a shift in pattern location. **e** A more complicated, two-dimensional, spatially restricted spot pattern can be formed by a combination of self-organization and instruction: the instruction step sets up positional information, which in turn influences self-organization. In this specific example, the gradient set up in the instruction step modulates the death rate of the gray cell type; yellow spots only form when this death rate is high enough (i.e., when *δ* is sufficiently high). For the combination mechanism, changes in instruction can globally expand or contract the region of spot formation, while changes in self-organization can increase the density of spots or turn spots into stripes.

Pattern variation (Fig. 1) arises from changes in developmental parameters that govern the dynamics of self-organization and instruction (e.g., the rate of cell migration or the shape of a morphogen gradient, Fig. 3a) or the interactions between them. Changes to developmental parameters can have multiple origins (Fig. 3b). For example, in some cats^41^ and snakes^19^, mutations in the coding regions of proteins that influence pigment cell behavior underlie specific pattern variants. Developmental parameters can also be affected by genetic changes in regulatory sequences or altered epigenetic states, which impact gene expression or translation^72,73^ and thereby migration, cell–cell interaction, proliferation or differentiation. This may, for example, be the case in striped mice, which have changes in regulatory elements surrounding patterning genes that do not occur in closely related unstriped mice^30^. Finally, developmental processes can be modulated by environmental factors^73,74^. For example, in clownfishes, the presence of adult conspecifics speeds up the death of pigment cells, which is required for the loss of white bars during maturation. This process is possibly mediated by thyroid hormone^75^.

An important challenge for the field is to predict how changes in developmental parameters impact pattern organization. Regardless of the causal factors underlying the change in pattern development, the resulting consequences for pattern organization will crucially depend on how the dynamics of self-organization and instruction (or the interactions between the two) are altered. Indeed, as we will propose below, self-organization and instruction contribute to the generation of pattern variation in qualitatively different ways.

As a toy example, we consider a hypothetical one-dimensional pattern that consists of three equally spaced domains (Fig. 3c, d). Such a simple pattern can be generated by a wide range of developmental mechanisms: here, for illustrative purposes, we consider two extreme possibilities. First, the pattern may result from the interactions between two types of motile pigment cells, whose interactions cause them to organize into alternating domains (self-organization; Fig. 3c). Second, the pattern may result from the interpretation of an exogenous morphogen gradient (instruction; Fig. 3d). For either mechanism, we can now predict what changes in pattern organization can result from changes in the underlying developmental parameters.

For the self-organization mechanism in Fig. 3c, we find that changes in pattern development can alter various pattern characteristics. For example, stronger auto-stimulation of the green cell type would lead to wider domains, whereas a faster-migrating gray cell type would lead to fewer domains. However, any changes would maintain the equal spacing of the domains and impact each domain similarly (Fig. 3c). The changes in pattern organization that are accessible under the instruction mechanism in Fig. 3d are qualitatively different. It is now possible to alter one of the three domains, while leaving the others unaffected, by leaving the morphogen gradient intact but changing the thresholds that define the domains. It also becomes possible to shift the pattern as a whole. However, because each domain is specified independently, a much more complex genetic rewiring that forms new thresholds would be needed in order to gain additional domains.

We hypothesize that the differences in variant patterns that can be obtained under self-organization or instruction in this toy example reflect general principles of self-organized and instructed pattern formation. Because self-organization relies on local interactions, developmental changes will typically alter the shape and spacing of each individual pattern element, which is determined locally; changes in local spacing can, in turn, dramatically alter the overall number of pattern elements. However, self-organization mechanisms would be much less likely to give rise to variants where individual pattern elements are impacted differentially (e.g., only one domain becoming wider). On the other hand, because pattern elements are specified independently under instruction, substantial increases in the number of pattern elements are unlikely. However, instruction facilitates pattern variation where individual pattern elements are altered (or disappear altogether) while others are left intact.

While patterns generated through pure self-organization (without any guiding instructive cues) or pure instruction (without any local interactions) are theoretically possible, most real-world patterns that have been studied have been found to combine self-organization and instruction^6,22,30,39,69^. For such combination mechanisms, we can think of self-organization and instruction as presenting different “dials” for pattern evolution, where the variation produced depends on the dial tuned. For example, for the spatially restricted spot pattern in Fig. 3e, the local organization of the pattern can be varied by tuning the self-organization dial—allowing for an increased density of spots or a shift from spots to stripes—whereas the size of the domain for pattern formation is under control of the instruction dial. Generally speaking, we hypothesize that self-organization controls the local organization of the pattern (i.e., density and shape of pattern elements) while instruction constrains its global location and orientation.

The proposed hypotheses about the contributions of self-organization and instruction to pattern variation, motivated by the examples in Fig. 3c–e, can be tested theoretically with a systematic investigation of the accessible pattern variation under different models of pattern formation (see Box 1). This comparison can be made at the level of self-organization vs. instruction, as in Fig. 3, but also at a more granular level, by comparing different mechanisms for self-organization and instruction. For example, self-organization can occur via different mechanisms (e.g., molecular, cellular, mechanical mechanisms^53^; single-vs. multi-component Turing systems^76^), and future work could elucidate to what extent these mechanisms differ in the types of pattern variation they will produce under small changes in developmental parameters. Perhaps there are certain types of pattern variation that can only feasibly be achieved under specific developmental mechanisms. It will also be interesting to see to what extent developmental mechanisms differ in their robustness, i.e., how much pattern organization tends to change when developmental parameters are varied.

Box 1 Toward mathematical models of color pattern evo-devo Mathematical models for pattern formation (reviewed in refs. ^60,119,120^) are indispensable for making accurate predictions for how changes in pattern development impact pattern organization. Here, we briefly outline different modeling approaches and identify areas for future theoretical work.

The most concrete theoretical models are agent-based and describe the behavior of individual cells with explicit rules. For example, a model for zebrafish pattern formation may stipulate that “melanophores are repulsed by xanthophores at rate *x*” or “xanthophores are *p*% more likely to die when the local density of melanophores increases by *y.*”^61,121^ Because agent-based models can directly incorporate experimental evidence, they are powerful tools to disentangle the contributions of different cellular behaviors to pattern formation. However, they can only be feasibly deployed when the cellular mechanisms of pattern formation have been investigated in detail.

There also exist more abstract pattern formation models whose goal is to develop a general understanding of principles of pattern formation that can apply across different systems. For example, the reaction-diffusion models pioneered by Alan Turing consist of equations

$$\frac{\partial u}{\partial t}=f\left(u,v\right)+{D}_{u}\cdot \frac{{\partial }^{2}u}{\partial {x}^{2}},\,\frac{\partial v}{\partial t}=g\left(u,v\right)+{D}_{v}\cdot \frac{{\partial }^{2}v}{\partial {x}^{2}}$$

that describe how two quantities u and v vary across time and space, as a result of movement (represented in the equations by the diffusion terms with coefficients ${D}_{u}$ and ${D}_{v}$) and local interactions (represented by f and g). In Turing’s original formulation^46^, u and v are interpreted as the concentrations of two diffusible molecules, but they could also represent, for example, the densities of different types of pigment cells. In a typical use case, the equations are chosen to capture some known aspects of the biology qualitatively, such as the capacity for self-organization (e.g., as evidenced by perturbation experiments^55^), local activation coupled to long-range inhibition^50^, or self-organization downstream of instructive signals^30^. Because the precise mathematical functions chosen are largely arbitrary, less biological information is required to deploy these models. Luckily, even though they are not fully specified by biology, the qualitative predictions of abstract pattern formation models are often remarkably robust^60,96^.

Both types of pattern formation models can be used to study pattern variation and evolution. Agent-based models will be most useful in systems where the molecular and cellular mechanisms of pattern formation are understood in detail, and can be deployed to understand quantitatively how the patterns of mutants or closely related species come about from changes in cellular behavior^61^. By contrast, more abstract models are useful when the developmental biology is less resolved or when the goal is to make qualitative predictions about pattern variation across a wider range of species, not all of which are empirically accessible. In such cases, the simplicity and parsimony of a more abstract model outweigh their lack of biological specificity.

Beyond specific empirical systems, pattern formation models also provide an interesting study object for evolutionary theory in their own right, exemplifying the developmental control of a complex phenotype. However, the evolutionary properties of these models are not well resolved. An accessible first step would be to investigate the possible evolutionary trajectories of simple self-organized patterns under changes in pattern development, both neutrally and under selection. While these evolutionary dynamics may, to some extent, depend on the specific self-organization model chosen, it is also likely these models have some general “evolutionary” features (such as labyrinthine patterns being “intermediate” between dark and light spot patterns; see Box 2). Progress on this front will enable theoreticians to use pattern formation as a model for conceptual questions in evolutionary developmental biology, for example, about how developmental systems achieve robustness, redundancy, or evolvability.

### Linking pattern development and pattern variation in practice

The examples in the previous section illustrate how the accessible variation in pattern phenotype is shaped by the underlying developmental mechanism. This idea has two important practical consequences. The first is that observations of pattern variation across different scales (Fig. 1) may hold clues to the underlying developmental mechanisms. The second is that insights into pattern development may inform testable predictions about how development constrains pattern variation and evolution. We explore both implications in the next two sections below. First, we take the perspective of a developmental biologist seeking to identify the mechanistic basis of a particular pattern, and we consider how their hypotheses and experiments can be informed by observations of pattern diversity. Second, we take the perspective of an evolutionary biologist seeking to explain the scope of pattern diversification, and we consider how they can investigate the role of development in shaping pattern diversity.

### Formulating developmental hypotheses

Despite major advances over the past decade, identifying the molecular mechanisms of pattern formation remains experimentally demanding. As a result, the number of organisms where we have a well-resolved molecular understanding of color pattern development is (and will remain) limited. On the contrary, data on color pattern variation is readily available and can be quantified using an ever-expanding pattern quantification toolbox (Box 2). Multiple aspects of this variation can be informative about the underlying developmental mechanisms.

First, some patterns are highly conserved within but vary across species (Fig. 1a), whereas other patterns vary within populations (Fig. 1b) or even within individuals (Fig. 1c). The scale at which pattern variation manifests likely reflects the relative contributions of genetic and non-genetic mechanisms. Genetic changes have been repeatedly found to underlie pattern variation both between and within species^6,77,78^. On the other hand, non-genetic factors, including environmental factors, are known to regulate temporal changes in color (e.g., between light and dark morphs) and pattern (e.g., ontogenetic shifts in stripe number) within individuals^73,75,79,80^. In some cases, environmental factors also influence pattern differences between individuals. For example, in American alligators, stripe number is positively correlated with the temperature at which the egg was incubated^81,82^. However, there are, to the best of our knowledge, no examples of environmental factors or non-genetically heritable changes underlying color pattern variation between species. Pattern variation between species is therefore most likely caused by genetic changes.

Second, pattern variation can be discrete or continuous. Some patterns exhibit discrete polymorphisms, as in snakes, which can be striped or ringed (e.g., Fig. 1b-ii)^83,84^; lynxes, which can be spotted, rosetted, or uniform^85^; or cheetahs, some of which have a blotched “king cheetah” pattern^86^. Such discrete variation is often subject to Mendelian inheritance and can be controlled by single genes or mutations^19,86,87^. Genetic variants can be either coding^39^ or non-coding, often mediated by a gene expression threshold that induces the transformation from one pattern into the other (referred to as a threshold trait)^88,89^. In contrast, other patterns vary more continuously, such as blotch patterns in poison dart frogs (e.g., Fig. 1b-i), which change continually over time^90^; ornament distribution in guppies^91^; and bar number in cichlids^92^. These types of continuous variation are generally found to have polygenic bases^91,92^.

Third, pattern variation is typically restricted to certain aspects of pattern organization. For example, the patterns of emperor angelfish change during ontogeny in terms of their shape (from concentric rings to stripes) and color (from white to yellow), but the consistent spacing between pattern elements remains largely unchanged (Fig. 1c-iii). Similarly, estrildid finches exhibit extensive variation in their plumage coloration and within-feather micro-patterning (e.g., spots vs. stripes vs. chevrons), but the boundaries between domains with different colors and micro-patterns are consistent between species^44^. We propose that these variable and consistent features hold clues to the relative contributions of self-organization and instruction during pattern formation.

To further develop this argument, we consider vertical bars, which are widespread in squamates and ray-finned fishes^43^ but whose developmental basis remains poorly understood. An understanding of the genes and developmental mechanisms underlying these patterns is only beginning to emerge from work in *Danio* species (i.e., relatives of the horizontally striped zebrafish), cichlids, and clownfishes^22,33,92–94^.

**Fig. 4:**
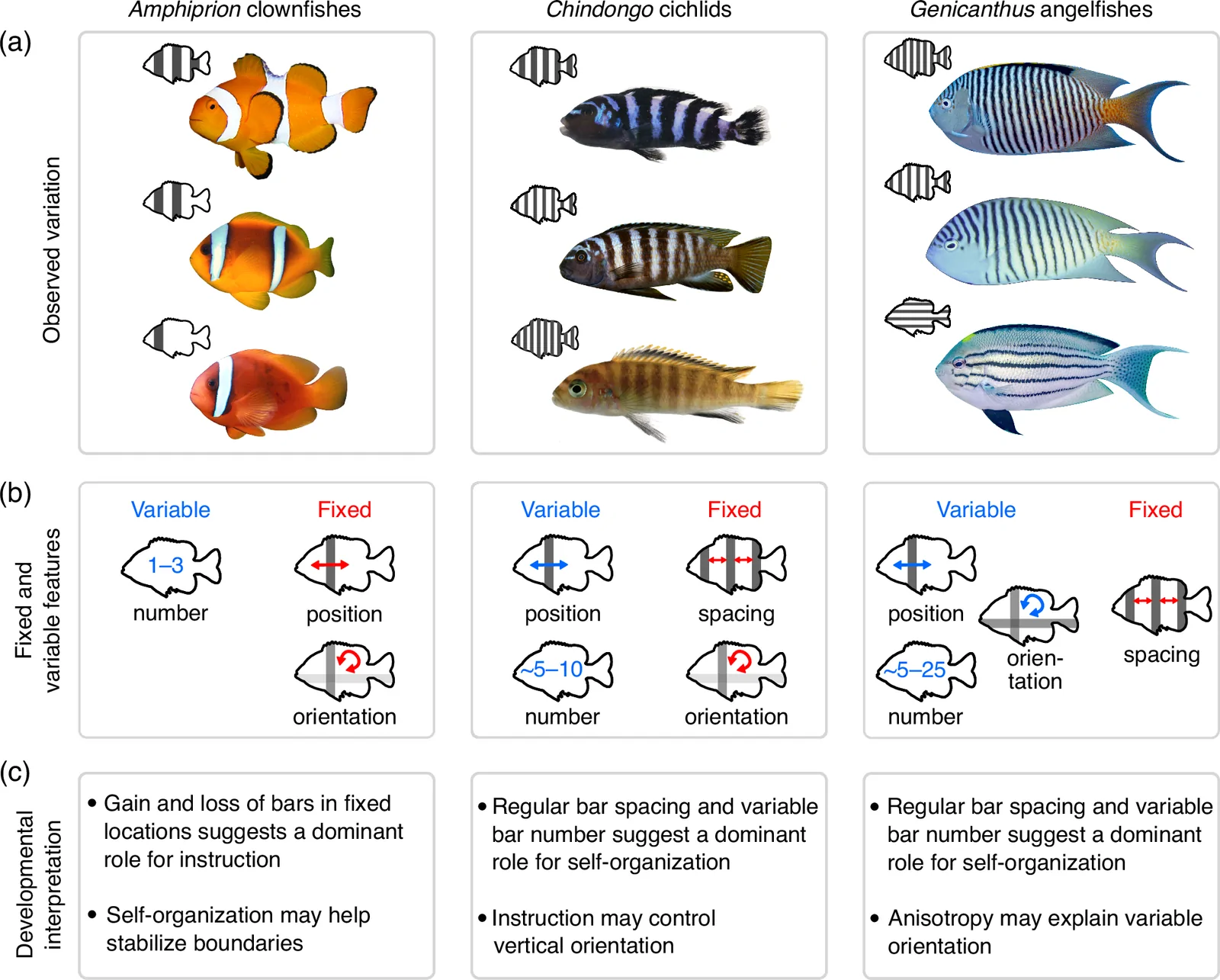
Developmental interpretations of pattern variation. **a** Examples of bar pattern diversity in three genera of fishes. Species shown (by genus, from top to bottom): *A. ocellaris* (photo credit N. Hobgood, CC-BY-SA), *A. bicinctus* (P.W. Jacobson, CC-BY), *A. frenatus* (B. Gratwicke, CC-BY), *C. demasoni* (M. Kuwalekar), *C. elongatus* (D. Midgley, CC-BY-SA), *C. flavus* (C.F. Kratochwil), *G. caudovittatus* (R. Vasconcellos, CC-BY), *G. melanospilos* (D. Baker, CC-BY), and *G. lamarck* (D. Delso, delso.photo, CC-BY-SA). Copyright licenses: https://creativecommons.org/licenses/by/4.0/, https://creativecommons.org/licenses/by-sa. We removed backgrounds and aligned fish horizontally. **b** Fixed and variable features of bar patterns. **c** Developmental hypotheses informed by the observed variation.

Although bars are common, taxa exhibit different patterns of bar variation (Fig. 4). For example, *Amphiprion* clownfishes have one to three white bars on a dark background, whose location is consistent even among species with different numbers of bars^33^: the first bar is always on the head, the second bar is on the trunk and centered on the spiny- to soft-ray dorsal fin transition, and the third bar is located on the base of the caudal fin. In African Mbuna cichlids of the genus *Chindongo* and angelfishes of the genus *Genicanthus*, the number of bars is higher and more variable, and bars vary in their anatomical location. Bar number can even vary within individuals as a result of branching, yet certain properties remain constant, including their regular spacing. The *Genicanthus* angelfishes also exhibit variation in orientation, as the genus contains horizontally striped fish as well.

A now testable hypothesis is that the observed differences arise because vertical bar pattern diversity is produced by qualitatively different developmental mechanisms across genera. The variation observed in clownfishes suggests a role for instructive cues in positioning bars; this would explain their fixed locations aligned with anatomical features and limited variability in number. On the other hand, variation in cichlids and angelfishes suggests a more prominent role for cross-species variation in self-organization dynamics; this would explain the large number of bars with variable locations but regular spacing. The striking shift in orientation in *Genicanthus* is also consistent with self-organized pattern formation and can be explained from subtle asymmetries in how local interactions take place along the body axes (i.e., anisotropy^95^).

Importantly, we expect pattern formation to generally incorporate both self-organization and instruction. In clownfishes, self-organization may help to define bar boundaries, because mutants with defects in cell–cell communication have malformed bars^39^. Nevertheless, even in these mutants, the overall pattern is maintained and remains symmetric across both sides of the same fish^39^, consistent with the bars being positioned by instructive signals. In cichlids, self-organization alone cannot explain the consistent vertical orientation. Instruction could account for this consistency^69,96^, for example, by controlling cell migration during development—a hypothesis supported by cell tracing studies in zebrafish, which show that pigment cells derived from single progenitors tend to be organized in vertical bands^97^.

The vertical bar example illustrates that considering pattern variation can inform hypotheses about how self-organization and instruction contribute to pattern development. Having such a hypothesis is helpful because it can help interpret molecular evidence and guide future experiments. The identification of the mechanisms underlying zebrafish pattern formation provides an example. While the zebrafish pattern of 4–5 horizontal stripes could a priori be generated in different ways, the pattern variation in related species and mutants—which include patterns with spots and labyrinthine patterns^13,22^—has been a strong indicator that self-organization maintains even spacing between the stripes. And this is indeed what was found experimentally: perturbation experiments confirmed that stripes are maintained by local interactions^55^. Subsequent empirical work has characterized the dynamic interactions between different types of pigment cells and shown that these cell–cell interactions met the criteria for a self-organizing system^22,38,56–59^. In another example, Haupaix et al. characterized the diversity of stripe patterns in different species of galliform birds and predicted that the position of yellow stripes would be under the control of an instructive signal^24^. Using long-term heterospecific skin grafts in which different parts of the skin tissue were transplanted between birds with different patterns, they then identified the source of this instructive signal, showing that yellow stripes are positioned by autonomous signaling from the somitic mesoderm (and then refined by local processes)^24^. Thus, the empirical identification of mechanisms of pattern development can be guided by hypotheses about the roles of instruction and self-organization; to formulate these hypotheses, pattern variation is much more informative than a single pattern in isolation.

Box 2 Approaches to quantifying color pattern variation Some of the approaches we discuss here require quantifying pattern variation in actual organisms. Existing quantification methods can be grouped into three categories: *descriptive* methods target the degree of similarity between patterns without considering ecology or development, *ecological* methods target whether patterns elicit similar responses in ecologically relevant viewers, and *developmental* methods target similarity in developmental mechanism. Below, we give a brief overview of these different approaches—emphasizing their differences and relative strengths—and identify a need for further work on developmentally informed methods for pattern quantification.

*Descriptive* methods characterize as much human- or machine-observable color pattern variation as possible, in the hopes of capturing the most salient aspects of variation. Methods in this category include adjacency analysis^122^ and recently developed R packages, such as *patternize*^123^, *recolorize*^15^, and *charisma*^124^. Typically, descriptive approaches begin by measuring many features (e.g., landmark-based alignment followed by color pattern segmentation) and then summarize them, for example, with principal components analysis (PCA)^125^. Because they work with RGB images and require little to no background information, descriptive methods can be applied across a wide range of datasets and taxa. Descriptive methods are well suited for describing variation in the size or intensity of conserved pattern elements (e.g., the chickadee patterns in Fig. 1a), but struggle with high-frequency periodic patterns like spots and stripes, where the number and exact locations of pattern elements are inconsistent across individuals (e.g., the frogs or snakes in Fig. 1b). For example, patterns with five and six evenly-spaced stripes would be considered dissimilar if the stripe locations do not overlap, even though those patterns may be developmentally and ecologically similar.

*Ecological* (or receiver-based) methods emphasize color pattern features that can affect behavior in an ecologically relevant receiver, such as a predator or potential mate. These include features used in early stages of visual processing, such as contrast, orientation, and frequency^17^. Tools in this category include the micaToolbox ImageJ plugin^126^, the related Quantitative Color Pattern Analysis framework^127^, the *pavo* package^128^, boundary strength analyses^129^, and receiver-based pattern morphospaces^34^. Ecological methods are excellent tools to address questions about color pattern function and sensory ecology, e.g., “Do birds perceive toxic frog species as more brightly colored than non-toxic ones?” Because ecological methods simulate non-human visual systems, they require knowledge about receiver ecology, visual systems, and behavior, and sometimes necessitate extra equipment (e.g., spectrometers or UV-sensitive cameras).

*Developmental methods* explicitly consider how a pattern develops and focus on features that are under direct control of developmental parameters; these are the features that are genetically encoded and can explain pattern differences across species. For example, for a self-organized pattern, the spacing of the pattern is likely developmentally controlled, whereas the location of individual pattern elements is less developmentally informative.

Developmental methods remain in their infancy compared to the recent proliferation of descriptive and ecological methods, in part because it is generally not obvious what features are developmentally informative. Empirically, these features could be identified by comprehensively analyzing pattern variation while controlling for genetic variation (e.g., by comparing patterns within individuals, Fig. 1c, or among genetically similar individuals), because at that scale developmentally informative pattern features would be expected to exhibit only limited variation. Theoretical modeling of pattern formation can also help to identify developmentally informative features: for example, self-organization models of zebrafish pattern formation have revealed statistical features of observed patterns (e.g., stripe curviness and variance in spot spacing) that can be predicted from the underlying cellular interactions^130^.

Developmental methods can be used to create developmental morphospaces whose axes are developmental parameters. Unlike descriptive and ecological morphospaces, traveling along these axes will produce realistic phenotypes at intermediate (unobserved) values. For example, Miyazawa^118^ used a developmental morphospace to correctly predict that the hybrid of a trout with dark spots and a trout with light spots would have a labyrinthine pattern (see Box Fig.); a descriptive, PCA-based approach would have incorrectly predicted a uniform intermediate pattern. Because of this ability to generate realistic hypothetical changes in pattern, developmental morphospaces will be broadly useful to reconstruct pattern diversification, test for developmental bias, and correlate pattern features with ecological variables^43,45,116,117^. Efforts in this area will therefore enable more eco-evo studies to account for the role of development in generating pattern diversity.

**Figure:**
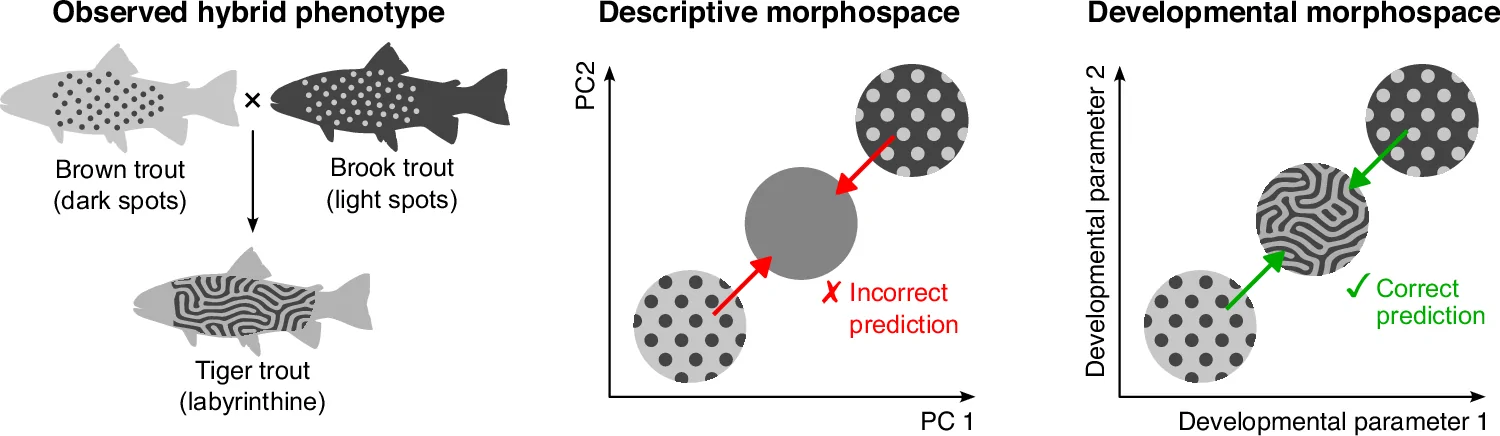
**Box Fig.**: A developmentally informed approach correctly predicts intermediate patterns.

### Testing for developmental biases

At a macroevolutionary scale, there are clear clade-specific trends in observed pattern diversity. For example, all squirrels have symmetric patterns of longitudinal stripes, but never transverse ones, while cichlid patterns generally consist of horizontal stripes, vertical bars, and apparent combinations thereof. Such general trends could result from selection for patterns with specific functions, for example, in camouflage^98^. Alternatively, they could reflect developmental biases^44,99,100^: if certain patterns are evolutionary more easily accessible as the mutational distance to reach them is shorter, then these patterns might be overrepresented even if they do not provide a selective advantage. Similarly, patterns that are observed only infrequently in specific clades may be rare because they are maladaptive and filtered away by selection, or because they are impossible or difficult to generate developmentally^101^. To distinguish between these hypotheses, it is critical to test whether observed trends in the phylogenetic distribution of patterns can be explained developmentally.

**Fig. 5:**
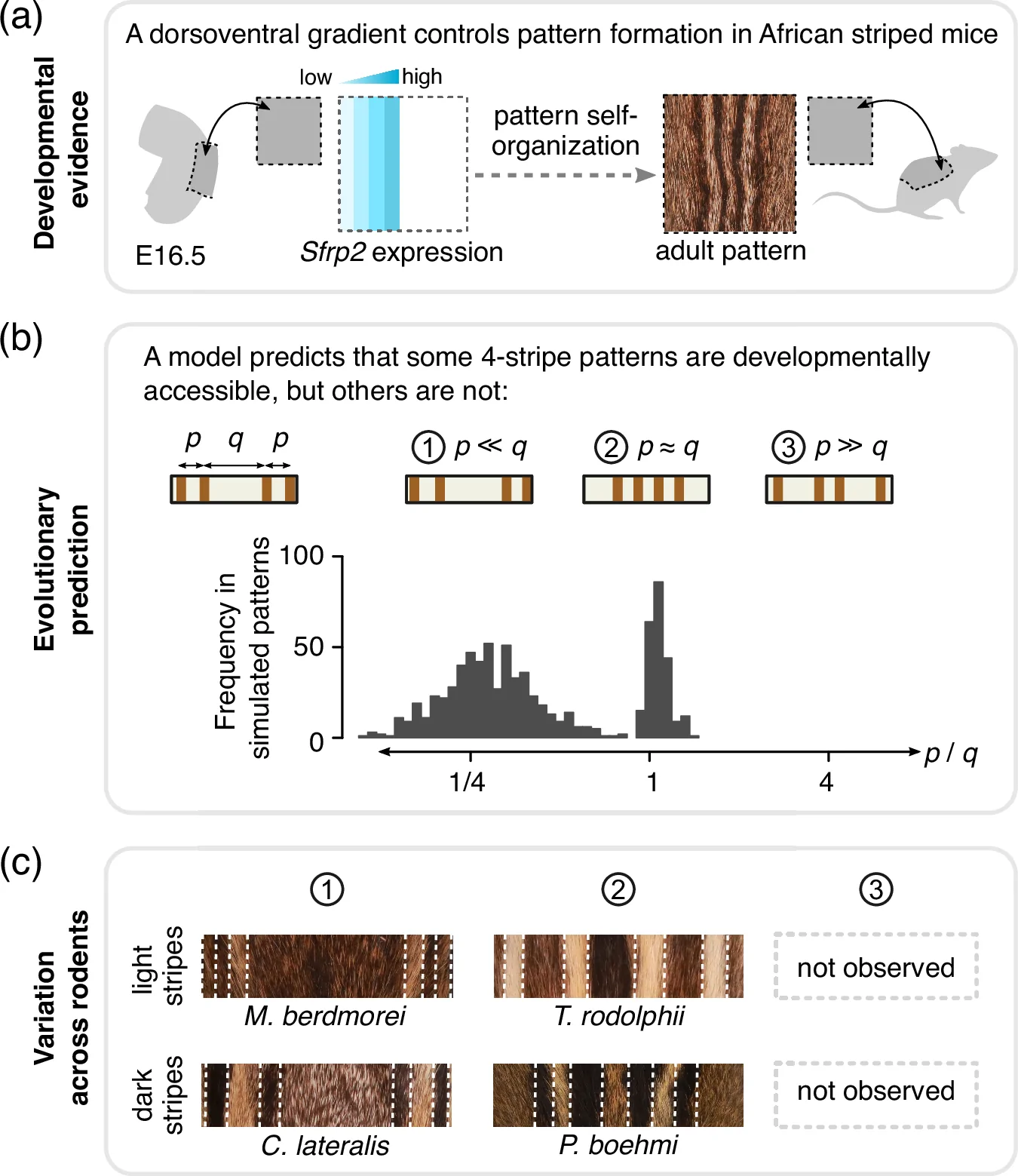
Making predictions about pattern variation based on developmental observations. **a** In African striped mice (*Rhabdomys pumilio*), an expression gradient of the gene *Sfrp2* guides pattern self-organization. **b** A developmental model based on the observed mechanism predicts that four-stripe patterns consisting of a pair of lateral stripes (pattern 1) or regularly spaced stripes (pattern 2) are possible, but a pattern with a pair of medial stripes and a lateral stripe on each side (pattern 3) is not. **c** Only patterns 1 and 2, but not pattern 3, have evolved in rodents. Figure adapted from ref. ^45^.

Testing whether and how development biases diversification requires a framework for making developmentally informed evolutionary predictions. Outside of color patterns, this has been done successfully by creating bespoke developmental models that capture phenotypic variation with only a few developmental parameters (e.g., shapes of bird beaks^102,103^, teeth^104–106^, and choanoflagellate colonies^107^). A similar approach can be used for color patterns, which have the additional advantage that there already exists an extensive toolbox of pattern formation models (Box 1) that can be tailored to the developmental specifics of particular clades^45,108,109^. Recent work on rodent stripe patterns illustrates this approach (Fig. 5). While most rodents have uniformly brown or gray coats, various squirrels (Fig. 1a) and mice have evolved stripe patterns that exhibit cross-species variation in their number, color, and relative positioning. The developmental mechanisms underlying rodent pattern formation have been studied in detail in only one species: the African striped mouse *Rhabdomys pumilio*^29,110^. The developmental evidence gathered from African striped mice suggests that its characteristic 4-stripe pattern is generated via a two-step mechanism in which positional information, in the form of a dorsoventral expression gradient of the Wnt modulator Sfrp2, instructs subsequent self-organization of the pattern (by an unknown molecular mechanism)^29,30^ (Fig. 5a).

To determine whether other rodent patterns could be generated via the same mechanism, a simple mathematical model was designed to capture the key developmental features—self-organization downstream of a dorsoventral gradient^45^. This model suggested that various stripe patterns with different numbers of stripes could be generated by changing the shape of the gradient, but also predicted developmental constraints on stripe positioning. In particular, out of all theoretically possible symmetric four-stripe patterns, only those consisting of two pairs of lateral stripes (pattern 1 in Fig. 5b) or of roughly equally spaced stripes (as in *R. pumilio*; pattern 2 in Fig. 5b) were developmentally accessible within the model’s parameter regime. By contrast, a hypothetical pattern consisting of a pair of medial stripes and a single lateral stripe on each side (pattern 3 in Fig. 5b) could not be generated^45^. A subsequent test of this prediction against the full set of evolved rodent stripe patterns confirmed the model’s prediction: no rodent has evolved the “inaccessible” pattern 3, while patterns 1 and 2 both have evolved multiple times (Fig. 5c). These findings thus suggest that, in rodents, the scope of pattern diversity can at least in part be predicted from developmental principles.

Even though rodent pattern formation relies on a combination of self-organization and instruction, the modeling work shows that variation in the instructive signals (i.e., the shapes of morphogen gradients) can recapitulate the evolved pattern diversity even without changes to self-organization parameters^45^. This raises broader questions about the relative roles of self-organization and instruction in pattern diversification. In other contexts, including tetrapod limb structure^111^, bird beak shapes^112^, and insect wing patterning^113^, phenotypic variation is often controlled by changes in instructive cues^5^. As the rodent example suggests, instruction may be key to color pattern diversification as well. Perhaps one reason why is that changes to instruction are more likely to generate adaptive variation than changes to self-organization. This hypothesis is supported by theoretical models indicating that self-organization parameters must be finely tuned to ensure pattern formation^114,115^, so that changes in these parameters often drastically disrupt the pattern or preclude pattern formation altogether. In contrast, changes in instructive cues allow for less dramatic changes in pattern and may thereby be more effective at producing variants that allow a gradual climb of an adaptive landscape. The hypothesis that instruction and self-organization contribute differentially to the generation of adaptive variation could be tested theoretically by in silico evolution of patterns under different selective regimes. Alternatively, the difference between self-organization and instruction may not lie in their ability to generate adaptive variation but rather in their pleiotropic consequences for other traits (as both instructive signals and mediators of cell–cell communication can affect features besides color pattern formation), a hypothesis that could be tested empirically by broadly phenotyping organisms with mutations in the involved genes.

Beyond dictating which patterns can and cannot evolve, development may also shape the phylogenetic distribution of pattern organization. A promising area of future work is to use models of pattern development to interpret phylogenetic data on pattern diversification^32,43,45,116,117^. Phylogenies of evolved patterns are notoriously difficult to interpret because patterns are highly dimensional traits that can vary drastically even between closely related species. The developmental morphospace of theoretically possible patterns, organized by their developmental similarity, is a powerful approach to formulate evolutionary predictions and disentangle the roles of selection and developmental biases in shaping pattern diversity (Box 2). Analysis of labyrinthine patterns in fish showcases the potential of this approach. Here, developmental models made the prediction that labyrinthine patterns can be interpreted as the result of the hybridization or “blending” of two spots patterns, one with dark spots on a light background and the other with light spots on a dark background^118^. Intriguingly, an exhaustive phylogenetic analysis of pattern diversity in fishes reveals that labyrinthine patterns are indeed more likely to evolve in clades where both types of spot patterns are present^43^. Thus, developmental insights can potentially help us understand not only the scope of pattern diversity but also macroevolutionary dynamics.

## Conclusion

In conclusion, in the 1980s, mathematical biologists established that intricate vertebrate color patterns, including zebra stripes and leopard spots, could be theoretically recapitulated by simple mathematical equations, suggesting that a small set of developmental principles suffices to explain much of the observed pattern diversity in nature. While this work was largely speculative at the time in the absence of molecular evidence, today we have strong developmental evidence that vertebrate pattern formation is indeed governed by shared developmental principles, including self-organization and instruction. This makes it possible to connect pattern development to pattern diversity both in theory and in practice. Crosstalk between development and diversity can be initiated from either direction: observations of cross-species diversity can inform developmental hypotheses, and, conversely, developmental insights can inform predictions about developmental biases that can be tested against the observed diversity. Mathematical modeling will continue to play a critical role, as models can now be tailored to the developmental mechanisms observed in specific clades and be used to formulate testable predictions about pattern variation and evolution. Altogether, an integrative approach that draws on mechanistic insights into pattern formation, theoretical models for pattern evo-devo (Box 1), and quantification of color pattern diversity (Box 2), can help us uncover the developmental “knobs and dials” of color pattern evolution and will help to establish vertebrate color patterns as a model system for evo-devo.
